# CGINet: graph convolutional network-based model for identifying chemical-gene interaction in an integrated multi-relational graph

**DOI:** 10.1186/s12859-020-03899-3

**Published:** 2020-11-26

**Authors:** Wei Wang, Xi Yang, Chengkun Wu, Canqun Yang

**Affiliations:** 1 College of Computer, National University of Defense Technology, Changsha, 410073 China; 2 State Key Laboratory of High-Performance Computing, National University of Defense Technology, Changsha, 410073 China

**Keywords:** Drug discovery, Chemical-gene interaction, Graph convolutional network, Integrated multi-relational graph

## Abstract

**Background:**

Elucidation of interactive relation between chemicals and genes is of key relevance not only for discovering new drug leads in drug development but also for repositioning existing drugs to novel therapeutic targets. Recently, biological network-based approaches have been proven to be effective in predicting chemical-gene interactions.

**Results:**

We present CGINet, a graph convolutional network-based method for identifying chemical-gene interactions in an integrated multi-relational graph containing three types of nodes: chemicals, genes, and pathways. We investigate two different perspectives on learning node embeddings. One is to view the graph as a whole, and the other is to adopt a subgraph view that initial node embeddings are learned from the binary association subgraphs and then transferred to the multi-interaction subgraph for more focused learning of higher-level target node representations. Besides, we reconstruct the topological structures of target nodes with the latent links captured by the designed substructures. CGINet adopts an end-to-end way that the encoder and the decoder are trained jointly with known chemical-gene interactions. We aim to predict unknown but potential associations between chemicals and genes as well as their interaction types.

**Conclusions:**

We study three model implementations CGINet-1/2/3 with various components and compare them with baseline approaches. As the experimental results suggest, our models exhibit competitive performances on identifying chemical-gene interactions. Besides, the subgraph perspective and the latent link both play positive roles in learning much more informative node embeddings and can lead to improved prediction.

## Background

Drug discovery is a complex, lengthy, inefficient, and expensive process. The estimated average time needed to launch a new drug is around 10–15 years at an average cost of about $1.8 billion [1]. To expedite the drug development process, it is critical to screen as many potential drug candidates as possible in the prophase. Over 80% of FDA-approved drugs are small-molecule chemicals that act on single or multiple gene (or protein) targets, ultimately achieving curative effects [2, 3]. Obviously, elucidation of interactive relation between chemicals and genes, named chemical-gene interactions (CGIs), is of key relevance not only for discovering new drug leads in drug development but also for repositioning existing drugs to novel therapeutic targets. With known CGIs, numerous researches provided new insights into rapidly screening candidate chemicals for treatments of corresponding diseases, such as HIV [4], HCV [5], lung cancer [6], and so forth. Unfortunately, proven CGIs are present in limited amounts. For example, the PubChem database contains more than 30 million chemicals, but few have confirmed gene targets [7]. This predicament drives the imperative need for automatic and efficient methods to infer chemical-gene interactions as a preliminary process rather than experimentally determining every possible chemical-gene pair, which is time-consuming and costly. According to different kinds of data used, we roughly divide the computational methods for CGI prediction into three categories: biomedical literature-based, molecular structure-based, and biological network-based.

### Biomedical literature-based approaches

A wealth of knowledge about chemical-gene interactions is scattered over the published biomedical literature, resulting in the inefficient query of CGI information of interest. The challenge is to detect the chemicals and the genes with close association mentioned in an unstructured text and further determine which type of interaction they share. Biomedical literature-based methods tackle the problems with well-designed or deep-learning features enhanced by natural language processing (NLP) techniques [8–10]. In recent studies, multiple deep neural network (DNN) models, including convolutional neural network (CNN), recurrent neural network (RNN), long short-term memory network (LSTM), and attention-based DNN, have been applied to learn CGI classifiers [11–13]. These approaches feed the DNN models with low-dimension pre-trained word embeddings without complicated feature engineering. Notably, attention-based DNN models exhibit competitive performance compared with other models and have the inherent ability to extract salient features for CGI identification as needed. Besides, some advanced researches extend the language models with syntax and semantic information, such as part of speech (POS), syntactic structure, dependency tree, and knowledge graph for a better understanding of the context [8, 14, 15]. However, such methods based on biomedical articles limit in predicting unpublished and unknown CGIs.

### Molecular structure-based approaches

Among these methods, molecular docking, which explores the predominant binding models of two interacting molecules using known 3D-structures, were initially studied [16, 17]. It uses various scoring functions to predict the binding affinity of molecules. The limitations lie in that it critically dependents on the available high-quality 3D-structure data and generally takes excessive computing resources. The follow-up researches focus on representing chemicals and genes by fingerprints as inputs of the machine-learning models [7, 18, 19], such as logistic regression, k-nearest neighbor (KNN), support vector machine (SVM), etc. Fingerprint is the most commonly used descriptor of the substructure of the molecule. However, the fingerprint is defined as a binary vector whose index value represents whether the substructure of a molecule exists or not, making it quite sparse and not sufficiently informative for CGI prediction. Recent researches have paid more attention to recruiting the end-to-end models on simplified molecular-input line-entry system (SMLES) string for chemicals and structural property sequence (SPS) for genes to learn super representations [2, 20–22]. The results achieved demonstrate that the models trained with super representations are more robust than those trained with traditional descriptors.

### Biological network-based approaches

Compared with molecular structure-based approaches, biological network-based approaches combine the chemical space and the gene space into a consistent space by a constructed heterogeneous network/graph. Chemicals and genes are treated as nodes of the network. The links between two nodes denote their interactive relations, including intra-domain relations between two nodes of the same type, e.g. chemical-chemical interactions, and cross-domain relations between two nodes belonging to different types, e.g. chemical-gene interactions [23]. Multiple large-scale databases have captured as much as possible of knowledge about chemical-gene interactions from the publicly accessible data, such as STITCH (Search Tool for InTeractions of Chemicals) [24], CTD (Comparative Toxicogenomics Database) [25]. The emergence of these aggregated databases provides new opportunities for CGI prediction. Numerous studies develop a slew of network-based inference models that integrate diverse CGI-related information from the heterogeneous network and automatically learn the features of individual nodes for predicting missing relations [26–28]. The biological network-based approach has excellent advantages in potential CGI extraction as it does not rely on specific biological properties description or 3D-structure data of molecules.

Research on identifying chemical-gene interaction is still in its infancy, and there is much room for improvement in its performance. In this manuscript, we present the CGINet model, using a framework of encoder-decoder, to formulate the CGI identification problem as a task of multi-relational link prediction between chemicals and genes in a heterogeneous network/graph containing three types of nodes: chemicals, genes, and pathways. CGINet employs the graph convolutional network (GCN) as an auto-encoder on aggregating, transforming, and propagating neighborhood information over the graph. We investigate two different perspectives on learning node embeddings. One is to view the graph as a whole, and the other is to adopt a subgraph view that initial node embeddings are learned with the binary association subgraphs and then transferred to the multi-interaction subgraph for final node embeddings learning. Lastly, the node embeddings are sent to the decoder, which uses a tensor decomposition model to formulate chemical-gene interactions. CGINet adopts an end-to-end way that the encoder and the decoder are trained jointly with known CGIs in a multi-relational graph.

We study three implementations of the CGINet models with various components and compare them with baseline approaches. As the experimental results suggest, our models exhibit competitive performances in predicting chemical-gene interactions. The main contributions of our work are: (1) We present a graph convolutional network-based model to predict the missing links between the chemicals and the genes in a heterogeneous graph. Our model takes advantage of the information from latent links based on biological insights, outperforming the baseline models. (2) The model which adopts a subgraph perspective can dramatically reduce the training time and also improves performance. (3) Our model is capable of predicting novel chemical-gene interactions, which are not appeared in the original graph.

## Results

### Experimental settings

We construct a multi-relational graph containing 65 types of chemical-gene interaction. Every given chemical-gene pair is identified into none, one or more interaction types. As most graph-based approaches have done [26–28], we randomly split the CGI instances into training, validation, and test sets for each interaction type, having 8:1:1 ratio. The CGINet model is optimized with an Adam optimizer [29], and the parameters used in our models are summarized in Table 1. We individually measure the performance of each interaction type using area under the receiver-operating characteristic (AUROC), area under the precision-recall curve (AUPRC), and average precision for the top-k identifications (AP@k). To avoid the overfitting issue, we perform cross-validation and initialize the trainable parameters with multiple random seeds. The experimental results are given as average performance. We implement the CGINet model with Python language using the Tensorflow package [30].

**Table 1:** The parameter used in our model

Parameter	Description	Value
$epoch$	The number of training epochs	10
$batch\_size$	The number of samples per training step	128
$d_{1} , d_{2}$	The embedding sizes in the total graph perspective	32, 16
$\overline{d}_{1} , \overline{d}_{2} ,\tilde{d}_{1} ,\tilde{d}_{2}$	The embedding sizes in the subgraph perspective	128, 64, 32, 16
$dropout$	The dropout rate	0.1
$lr$	The learning rate of the Adam optimizer	0.001
$m$	The margin value of the hinge loss function	0.1

We study three model implementations CGINet-1/2/3 with various components and compare them with baseline approaches (DeepWalk [31], Node2Vec [32], SVD [33], Laplacian [34], GCN [35]). Brief descriptions about these approaches are given as follow:

#### Baseline approaches

(1) Random walk-based embeddings. The DeepWalk model learns node embeddings by randomly capturing neighborhood information on the basis of the depth-first search method, while the Node2Vec model combines the depth-first search and the breadth-first search methods to aggregate proximal nodes. (2) Matrix factorization-based embeddings. The SVD and the Laplacian models both factorizes the adjacency matrix of the graph to obtain the node embeddings. We use these learned node embeddings as input to train a logistic regression classifier for each interaction type. (3) Graph convolutional network-based methods. We employ a 2-layer GCN on learning node embedding with the CG-graph or the total graph, respectively named as GCN-CG and GCN-Total.

#### CGINet-1/2/3/ approaches

CGINet-1, CGINet-2, and CGINet-3 all adopt a subgraph view of learning final node embeddings by two steps. Besides, CGINet-2 and CGINet-3 take account of encoding information across latent links. The latent rate $\mu$ in the CGINet-2 model is a trainable parameter ($\mu \in [\mathrm{0,1}]$), while it is fixed to the value of 1 in CGINet-3 ($\mu =1$).

### Performance comparison of different thresholds

**Fig. 1:**
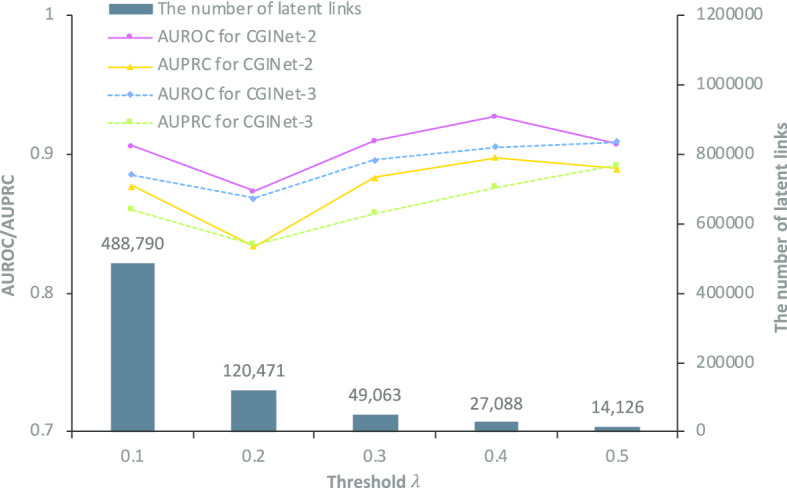
Performance comparison of different thresholds. The value of the threshold $\lambda$ ranges from 0.1 to 0.5 with step 0.1, as the threshold larger than 0.5 results in getting none of latent links for some interaction types, which we suggest that it is unfair

A threshold coefficient $\lambda$ is designed in our model as a gatekeeper to control the requirement of a definite latent link. We investigate the change of performance of our model with different thresholds. As shown in Fig. 1, Larger threshold leads to less latent links. The overall performance of CGINet-2 and CGINet-3 increases with the growth threshold. To be specific, CGINet-2 with $\lambda =0.4$ and CGINet-3 with $\lambda =0.5$, show respectively better performance. These suggest that stricter threshold value makes the latent links more credible for updating the topological structure of the graph. We proceed by making a performance comparison between the CGINet models with various components and baseline models.

### Comparison with baseline models

Table 2 gives the performance comparison of our models with baseline methods. Matrix factorization-based approaches and random walk-based approaches both learn node embeddings and train relation classifiers in two individual stages. The latter methods (SVD, Laplacian) show better performance than the former methods (DeepWalk, Node2vec) on processing such a heterogeneous multi-relational graph. Random walk-based approaches excessively dependent on the specific structure of the graph. In contrast, the CGINet models train the encoder and the decoder jointly. Most of our models outperform the baseline models, especially the CGINet-2 model achieves 5.7% of relative improvements in AUPRC compared with the best results of baselines (Laplacian).

**Table 2:** Performance comparison of our models with baseline approaches

Model	Component	AUROC	AUPRC	AP@20	TIME
DeepWalk	CG-graph	0.830	0.811	0.733	–
Node2Vec	CG-graph	0.819	0.800	0.735	–
SVD	CG-graph	0.833	0.823	0.772	–
Laplacian	CG-graph	0.839	0.841	0.765	–
GCN-CG	CG-graph	0.855	0.830	0.742	**2.2**
GCN-total	Total graph	0.823	0.768	0.571	8.5
CGINet-1	Two subgraphs	0.901	0.872	0.770	2.4
CGINet-2	Two subgraphs, $\lambda = 0.4$, $\mu \in \left[ {0,1} \right]$	**0.927**	**0.898**	0.765	2.9
CGINet-3	Two subgraphs, $\lambda = 0.5$, $\mu = 1$	0.914	0.893	**0.804**	2.8

The values of each metric are average performance in terms of different random seeds. The results are average performance values for all interaction types. TIME denotes the average training time of each epoch and it is measured in hours. The best result of each performance index is boldfaced

Compared to GCN-CG, GCN-Total shows manifest performance degradation. Especially it drops to 57.1% in AP@20. We hypothesize that the reason behind this is due to the limitation of the GCN-Total model in focusing on capturing interactions of interest in an integrated multi-relational graph that contains non-target associations (e.g. chemical-pathway associations, gene-pathway associations). Based on this assumption, we investigate a subgraph view of learning target node representations by two steps in the CGINet-1 model. It is inspiring to see that CGINet-1 outperforms GCN-Total by 7.8% (AUROC), 10.4% (AUPRC), and 19.9% (AP@20), indicating that more focused learning of node embedding facilitates better use of the graph data. Furthermore, compared with GCN-CG, CGINet-1 leads to about 4% of relative improvements in AUPRC. It verifies that initial node embeddings pre-trained with the binary association subgraph provide practical knowledge for final node embedding learning.

A further comparison among our models (CGINet-1, CGINet-2, and CGINet-3) reveals that the models which aggregate information from the new neighbor nodes across latent links perform better than the models only capture labeled neighborhood information. To be specific, CGINet-2 and CGINet-3 lead to about 2% increase in AUPRC compared with CGINet-1. It is consistent with our findings in “Data observation” section that updating the topological properties of nodes with latent links can significantly provide informative features for learning more effective node embeddings. Besides, CGINet-2 exhibits optimal performance in AUROC (92.7%) but is inferior to CGINet-3 by down to 76.5% in AP@20. In view of the overall situation, the latent rate setting enhances the classification power of the model but along with the poor ranking ability. Consequently, the CGINet-3 model, which considers the equal contribution of latent links for each interaction type, has better higher overall performance.

The above analysis has illustrated that our models which adopt the subgraph view can significantly improve performance. We also calculate the average training time of each epoch for the GCN-based models, as shown in the last column of Table 2. Compared with GCN-Total, our models can reduce at least 65% of training time while achieving much better performance.

### Comparison on interaction type-wise performance

**Fig. 2:**
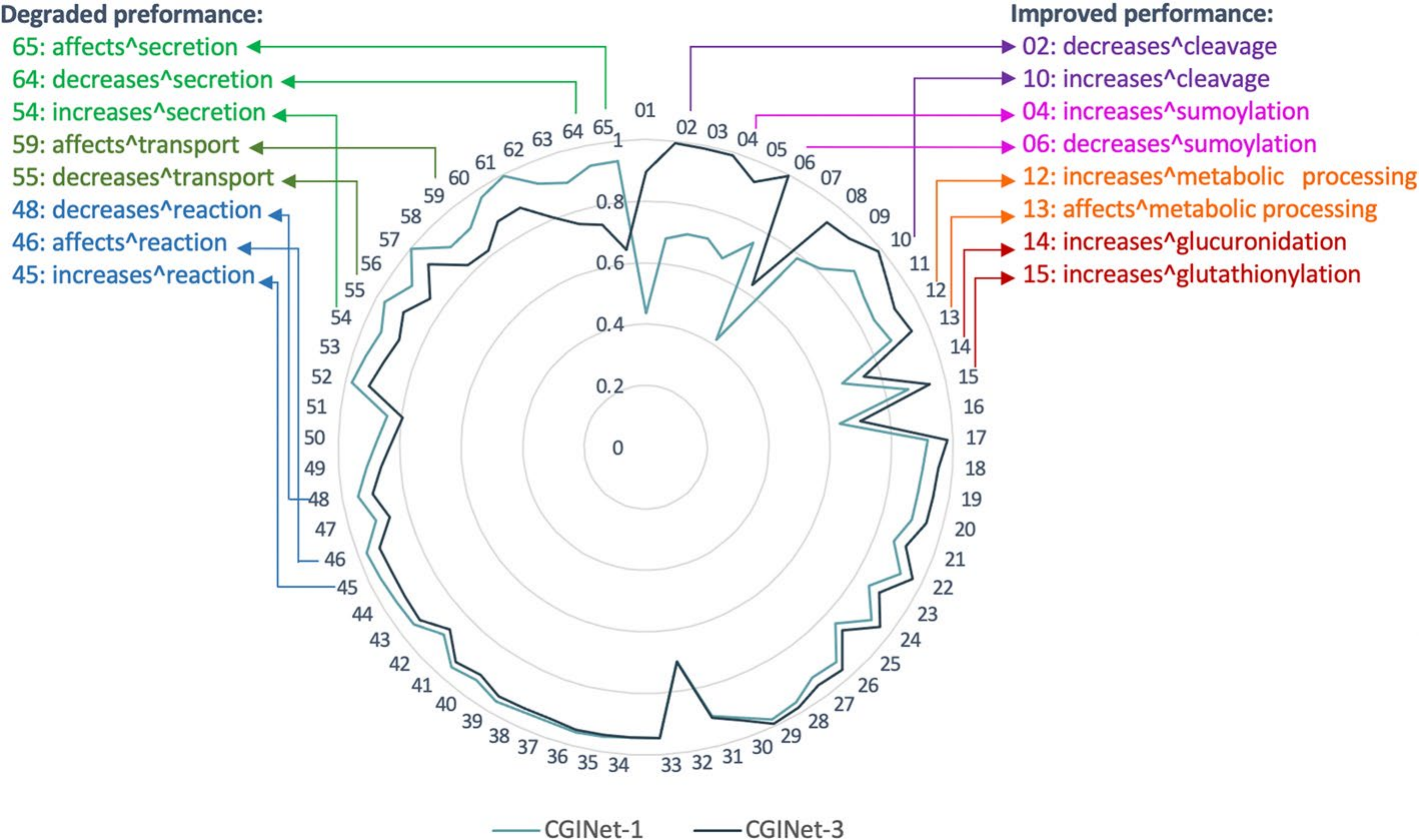
Comparison of CGINet-1 and CGINet-3 on interaction type-wise performance in AUPRC. The interaction type identifiers are sorted by the difference between the performances of CGINet-3 and CGINet-1 in AUPRC

As shown in Fig. 2, compared to CGINet-1, CGINet-3 achieves improved performances on over half interaction types (34 of 65 types; right side of Fig. 2) but gets degraded performances on the other types (left side of Fig. 2). Through detailed investigation on the performance per interaction type, we find that encoding updated neighborhood information across latent links prefers to play a positive role in predicting some specific interaction types without considering the degree of action (e.g. cleavage, sumoylation, metabolic processing, and glucuronidation), but participates negatively in identifying some other types (e.g. secretion, transport, and reaction). More interestingly, metabolic processing is the parent interaction type of cleavage, sumoylation, and glucuronidation. It inspires us to optimize our models by paying more attention to the deep-seated mechanism of the biological reaction in later research.

We visualize the top 15 best performance interaction types in the CGINet-3 model, as shown in Table 3. It is also worth noting that even though some interaction types have extremely few known edges for training, the model can still be adept at predicting them, e.g. decreases^acetylation (147 edges), affects^chemical synthesis (181 edges) and decreases^cleavage (188 edges). We believe that developing a global decoder associated with all interaction types enables our model to share information across different types of interactions.

**Table 3:** Top 15 best performance interaction types

Parent type	Interaction type	AUPRC	Edges
Metabolic processing	decreases^sumoylation	0.997	392
Metabolic processing	decreases^cleavage	0.993	188
–	increases^localization	0.991	918
Transport	decreases^uptake	0.989	266
Metabolic processing	increases^sumoylation	0.989	933
Metabolic processing	increases^cleavage	0.988	3872
Metabolic processing	affects^chemical synthesis	0.981	181
–	decreases^localization	0.980	224
Metabolic processing	decreases^acetylation	0.969	147
Metabolic processing	increases^degradation	0.966	1511
–	decreases^response to substance	0.960	5891
Metabolic processing	increases^phosphorylation	0.956	12,644
Metabolic processing	increases^chemical synthesis	0.955	6269
Metabolic processing	affects^phosphorylation	0.954	644
Metabolic processing	increases^ubiquitination	0.948	241

## Discussion

For the random walk-based approaches, the chemical space and the gene space are combined into a consistent space. The node embeddings are learned in a homogeneous graph. In contrast, the essence of our model is to analyze the dependency between different semantic spaces in a heterogeneous graph. It allows us to integrate more diverse biomedical data into our model, such as the disease and the phenotype information. We can not only explore the relation between chemicals and genes but also discover more internal connections in the molecular and patient population data.

It also worth noting that our model is capable of predicting novel chemical-gene interactions which are not appeared in the original graph. With Eqs. (4) and (5), we can calculate the probability ${\mathcal{P}}_{r}^{ij}$ of unknown chemical-gene pairs $({c}_{i},{g}_{j})$ under each interaction type $r$. Higher probability indicates that chemical ${c}_{i}$ inclines to interact with the gene ${g}_{j}$. We can turn to the online public databases to see whether or not the corresponding literature evidence can be retrieved. Table 4 provides some novel predictions with literature evidence.

**Table 4:** Novel chemical-gene interactions predicted by CGINet

Chemical	Gene	Prediction	References
Trantinterol	CYP2C9	Decrease activity	Jiang et al. [36]
Mepazine	KCNH2	Decrease activity	Slavov et al. [37]
Cuprizone	ADA	Increase expression	Abe et al. [38]
Tetrachlorodian	TFAP2A	Increase expression	Liang et al. [39]

## Conclusions

In this paper, we present CGINet, a graph convolutional network-based method for predicting compound-gene interactions in an integrated multi-relational graph. CGINet adopts a subgraph view that the initial node embeddings are learned with the binary association subgraphs and then transferred to the multi-interaction subgraph for more focused learning of higher-level target node representations. The experimental results have shown that the CGINet models exhibit competitive performance compared with the baseline models. Moreover, learning node embeddings with latent links can lead to improved performance.

CGINet is a transductive learning method that is applied to a static graph. To be specific, we train the graph neural network with all known nodes and part of edges (training edges) in the graph, producing node embedding for each node. The graph neural network learns the node embedding from neighborhood information through the adjacency matrix (or Laplacian matrix). That is to say, adding new nodes to the graph will change the adjacency matrix (or Laplacian matrix). The model should be retrained. This inherent property makes the graph neural network poor in dealing with the dynamic graph. In future work, we are interested in enhancing the capacity of our model for dealing with the dynamic graph. Moreover, we will gather more diverse biomedical information (e.g. compound-disease associations, gene-disease associations, and pathway-disease associations) and pay more attention to constructing a larger-scale bio-network for thoroughly analyzing the mechanism of action about the biological reactions. We aim to build a robust model for figuring out the long dependency between different molecules with better interpretability.

## Methods

### Integrated multi-relational graph

We construct a heterogeneous graph containing three types of nodes: chemicals, genes, and pathways, where pathway can shed light on the mechanism of action underlying CGI. A total of five individual chemicals/genes/pathways related graphs, including four binary association subgraphs [chemical-chemical graph (CC-graph), gene–gene graph (GG-graph), chemical-pathway graph (CP-graph), and gene-pathway graph (GP-graph)] and one multi-interaction subgraph [chemical-gene graph (CG-graph)], are collected from multiple curated databases and used to construct an integrated multi-relational graph.

#### Binary association subgraphs

We extract the CC-graph from the STITCH database, which contains 17,705,818 chemical-chemical associations across 389,393 chemicals. For the GG-graph, we grab 715,612 gene–gene associations between 19,081 genes complied by Decagon [40]. We obtain the CP-graph and GP-graph from the Comparative Toxicogenomics Database. There are 1,285,158 chemical-pathway associations and 135,809 gene-pathway associations consisted of 10,034 chemicals, 11,588 genes, and 2,352 pathways.

#### Multi-interaction subgraph

A link in the multi-interaction graph represents the association between two nodes as well as their interaction type. We construct the CG-graph by 13,488 chemicals, 50,876 genes, and 1,935,152 chemical-gene interactions pulled from the Comparative Toxicogenomics Database. Each CGI has a degree (increases, decreases, or affects) and type (e.g. activity, expression, and reaction), e.g. “Chemical X decreases the activity of Gene Y”, denoted as a triple (chemical X, decreases^activity, gene Y).

**Fig. 3:**
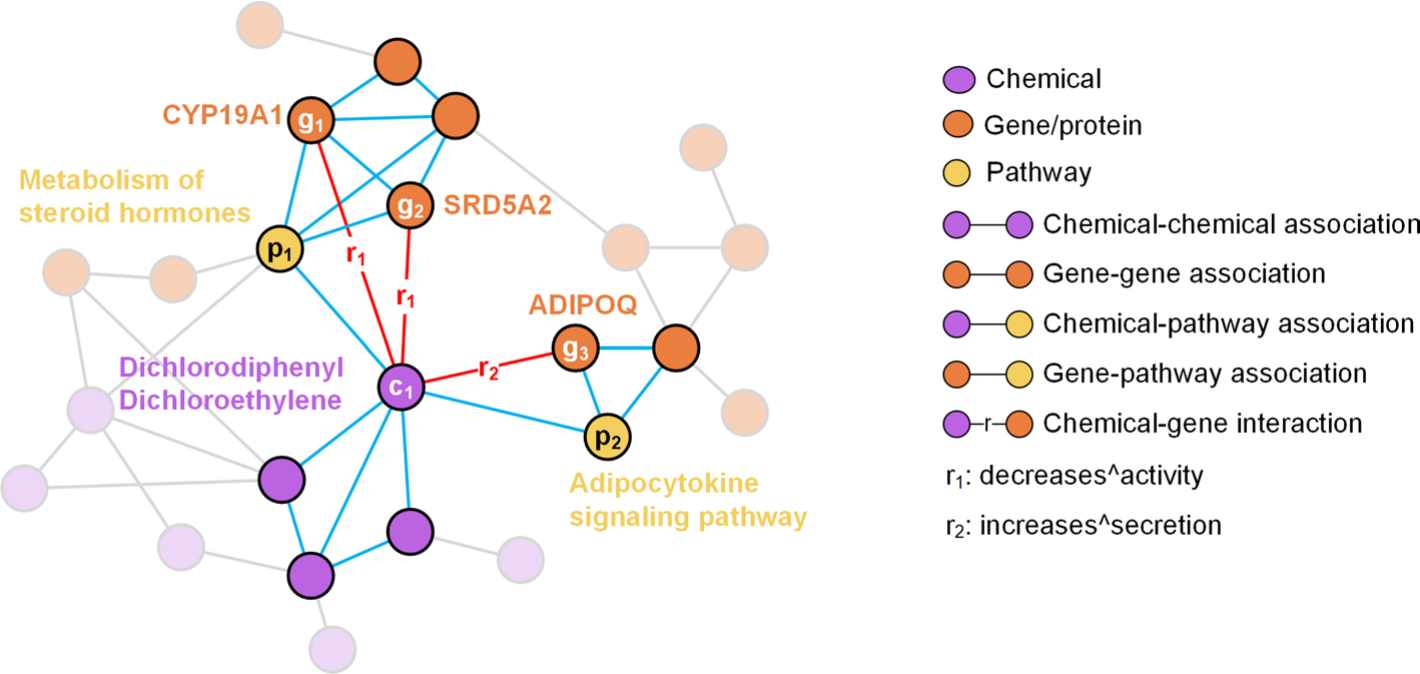
An example of the integrated multi-relational graph. The links shown in red indicate that Dichlorodiphenyl Dichloroethylene (node $c_{1}$) results in decreased activity of CYP19A1 (node $g_{1}$), SRD5A2 (node $g_{2}$), and increased secretion of ADIPOQ (node $g_{3}$). Chemical–chemical associations, gene–gene associations, chemical-pathway associations, and gene-pathway associations involved in this case are marked as highlighted blue links

Herein, we consider only 65 types of interactions between chemicals and genes that each appears in at least 180 CGIs. Besides, the CC-graph and the GG-graph are both trimmed by deleting nodes not involved in the CP-graph, GP-graph, and CG-graph. The final integrated graph has 14,269 chemicals, 51,069 genes, and 2,363 pathways. These nodes are connected by a total of 4,653,387 associations/interactions. An example of the integrated multi-relational graph and the detailed statistical data of the final graph are shown in Fig. 3 and Table 5, respectively.

**Table 5:** The detailed statistical data of the final integrated multi-relational graph

Subgraph	Association/interaction	Edges
CC-graph	Chemicals associate with target chemicals	720,155
GG-graph	Genes associate with target genes	713,469
CP-graph	Chemicals associate with target pathways	1,285,158
GP-graph	Genes associate with target pathways	135,809
CG-graph	Chemicals interact with target genes	1,798,796

### Data observation

**Fig. 4:**
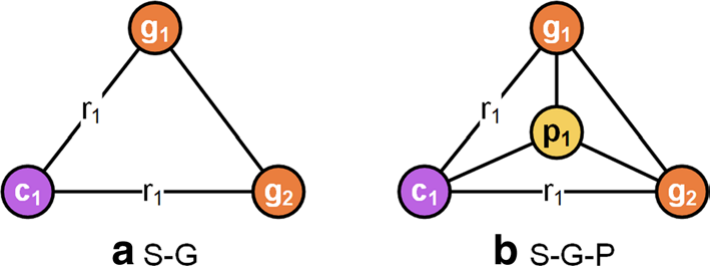
The examples of the S-G and the S-G-P. **a** S-G. Node $c_{1}$, $g_{1}$, and $g_{2}$ are linked in pairs. **b** S-G-P. Based on the structure of the S-G, chemical and gene nodes also share node $p_{1}$ in the S-G-P. The S-G indicates the potential interaction between chemical $c_{1}$ and gene $g_{2}$ if chemical $c_{1}$ interacts with gene $g_{1}$ which associates with gene $g_{2}$. Besides that, the S-G-P also considers the mechanism of action (pathway $p_{1}$) underlying the chemical-gene interaction and gene–gene association

The clustering result achieved in Parsons et al. [41] suggests that the chemicals incline to cluster with the genes related to each other. More specifically, if chemical ${c}_{1}$ interacts with gene ${g}_{1}$, and gene ${g}_{1}$ genetically associates with gene ${g}_{2}$, then we can reasonably assume that chemical ${c}_{1}$ and gene ${g}_{2}$ chemically genetically interact. In other words, there is a latent link connecting chemical ${c}_{1}$ and gene ${g}_{2}$. Based on this assumption, we carry on an observation about two types of topological substructures, S-G and S-G-P. Figure 4 gives examples of these two substructures.

**Fig. 5:**
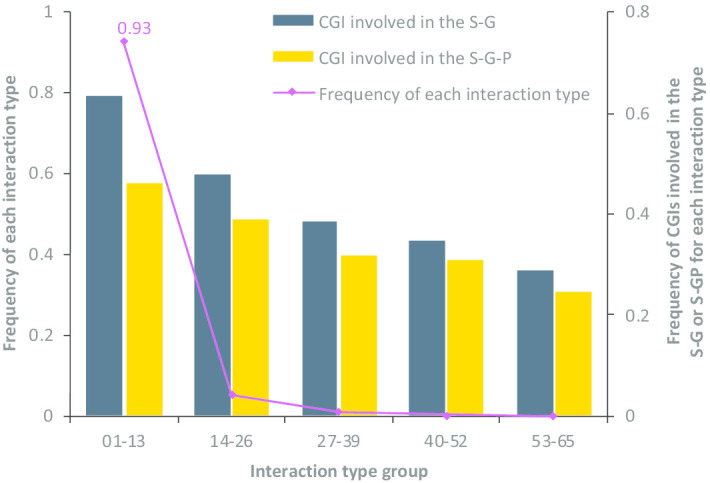
The frequency distribution of interaction types and the proportion of CGIs involved in the S-G or S-GP for each interaction type group. The interaction type identifiers are sorted by the number of CGIs for each interaction type. 65 interaction types are stratified into five groups: the interaction type identifier from 01 to 13, 14 to 26, 27 to 39, 40 to 52, and 53 to 65

Firstly, the substructures matched with the S-G and the S-G-P are extracted separately from the entire multi-relational graph. Secondly, we respectively count the number of CGIs that existed in the S-G or the S-G-P with de-duplication. After that, we investigate the frequency distribution of interaction types and the proportion of CGIs involved in the S-G or the S-G-P for each interaction type. We find that: (1) averagely, > 62% of individual CGIs are involved in the S-G, and about 50% of individual CGIs are involved in the S-G-P, suggesting that it is significant to capture unknown but potential links to update the topological properties of chemicals and genes for learning much more informative node embeddings. (2) The frequency of CGIs involved in the S-G or the S-G-P both decrease with the reduction of the total number of CGIs for each interaction type group (Fig. 5). The reason probably lies in the extreme imbalance of data, where 20% of interaction types capture about 93% of CGIs (e.g. increases^expression, decreases^expression, and affects^cotreatment). Therefore, we make a specific investigation on whether or not different contributions of latent links for each interaction type should be considered in “Results” section. These findings have remarkable inspirations for the development of the model in the following section.

### Problem formulation

**Fig. 6:**
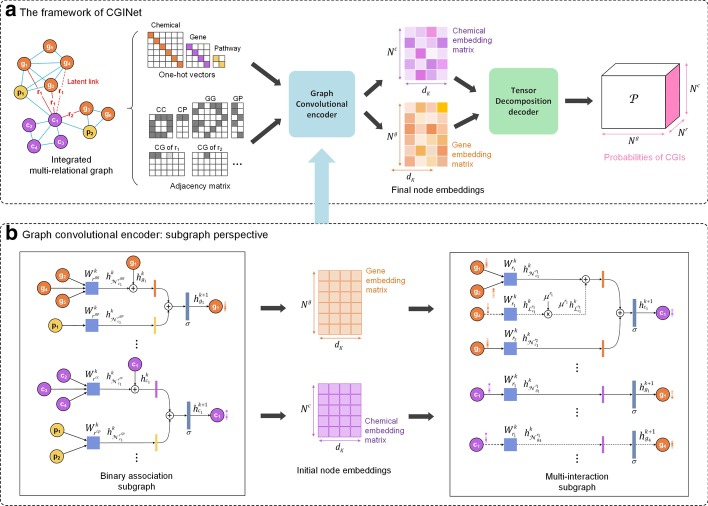
The flowchart of the CGINet pipeline. **a** The framework of CGINet. The graph convolutional encoder takes the integrated multi-relational graph as input (the one-hot vectors for each node and the adjacency matrices) and returns a chemical embedding matrix and a gene embedding matrix. The tensor decomposition decoder uses these node embeddings to compute the probabilities of interactions between the chemicals and the candidate genes. **b** Graph convolutional encoder. We take the subgraph perspective as an example. Initial embeddings of chemicals $c_{1}$ and genes $g_{1}$ are learned with the binary association subgraph. For example, $c_{1}$ receives information from neighbor nodes, including chemical nodes ($c_{2}$, $c_{3}$, $c_{4}$) and pathways ($p_{1}$ $p_{2}$). The initial embeddings are then transferred to the multi-interaction subgraph for learning final embeddings. In the multi-interaction subgraph, the encoder aggregates information not only from the neighbor nodes across known edges but also from the new neighbors connected by latent links (shown in dotted line). For example, $c_{1}$ encodes neighborhood information from $g_{1}$, $g_{2}$, $g_{3}$ and $g_{4}$

**Fig. 7:**
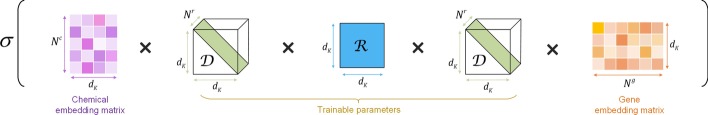
Tensor decomposition decoder. The chemical embedding matrix and the gene embedding matrix are learned from the graph convolutional encoder. Tensor ${\mathcal{D}}$ is a set of the matrix ${\mathcal{D}}_{r}$

The CGI identification problem is formulated as a task of link prediction in the integrated multi-relational graph including four binary association subgraphs and one multi-interaction subgraph. We denote the associated relation set as ${\stackrel{-}{R}=\{r}^{cc},{r}^{gg},{r}^{cp},{r}^{gp}\}$, and the interactive relation set as $\stackrel{\sim }{R}={\{{r}_{i}^{cg}\}}_{i\in {[N}^{cg}]}$, where ${N}^{cg}$ is the number of interaction types. Given a set of chemicals ${V}_{c}={\{{v}_{i}\}}_{i\in [{N}^{c}]}$, a set of genes ${V}_{g}={\{{v}_{i}\}}_{i\in [{N}^{g}]}$, and a set of pathways ${V}_{p}={\{{v}_{i}\}}_{i\in [{N}^{p}]}$, where ${N}^{c/g/p}$ is the number of chemicals/genes/pathways, the entire graph can be denoted as $G=(V,E)$, where $V=\{{v}_{i}|{v}_{i}\in {V}_{c}\cup {V}_{g}\cup {V}_{p}\}$ and $E=\left\{\left({v}_{i},r,{v}_{j}\right)|r\in \{\stackrel{-}{R}\cup \stackrel{\sim }{R}\}\right\}$. Using the graph $G$, our goal is to calculate the probability of an edge ${e}_{ij}={\left({v}_{i},r,{v}_{j}\right)}_{i\in [{N}^{c}],j\in [{N}^{g}]}$ of interaction type $r$ be assigned to $\stackrel{\sim }{R}$, which implies that how likely chemical ${v}_{i}$ results in an interaction type $r$ of gene ${v}_{j}$. To achieve that, we develop an end-to-end trainable model CGINet (Fig. 6a) that has two main components, a graph convolutional encoder (Fig. 6b) and a tensor decomposition decoder (Fig. 7).

### Graph convolutional encoder

Much research has proved graph convolutional networks to be effective in node/graph representation learning [42, 43]. The graph convolutional network usually extracts local substructure features for individual nodes by iteratively aggregating, transforming, and propagating information from neighbor nodes. A deeper graph convolutional network can integrate the normalized message from all neighbors up to k-hops away. Notably, 2-layer graph convolutional network models yield the best performance based upon empirical observation [44].

Herein, we propose an encoder equipped with 2-layer graph convolutional networks taking the graph $G$ as input and producing topological-preserving embedding ${z}_{i}$ for each node. We investigate two perspectives on encoding neighborhood information with the graph $G$: total graph perspective and subgraph perspective. The former is to view the graph as a whole, while the latter is to adopt a subgraph view that initial node embeddings are learned with the binary association subgraphs and then transferred to the multi-interaction subgraph for final node embeddings learning.

#### Total graph perspective

A 2-layer graph convolutional network operates directly on the entire multi-relational graph $$G$$. In each layer, GCN updates the embedding for each node by simply summing different nearby information propagated across different types of edges. Given the $$k$$th hidden state $$h_{i}^{k}$$ of node $$v_{i}$$, where $$v_{i} \in \{ V_{c} \cup V_{g} \cup V_{p} \}$$, the ($$k + 1)$$th hidden state $$h_{i}^{k + 1}$$ of node $$v_{i}$$ is specifically updated as follow:

1 $$h_{i}^{k + 1} = \sigma \left( {\mathop \sum \limits_{r} \mathop \sum \limits_{{j \in {\mathcal{N}}_{i}^{r} }} \frac{1}{{\sqrt {\left| {{\mathcal{N}}_{i}^{r} } \right|\left| {{\mathcal{N}}_{j}^{r} } \right|} }}W_{r}^{k} h_{j}^{k} + \frac{1}{{\left| {{\mathcal{N}}_{i}^{r} } \right|}}h_{i}^{k} } \right),$$

where $h_{i}^{k} \in {\mathbb{R}}^{{d_{k} }}$ with $d_{k}$ denotes the embedding size of the $k$th hidden layer. $r \in \left\{ {\overline{R} \cup \tilde{R}} \right\}$ denotes one of the interaction types. $W_{r}^{k}$ is the trainable parameter matrix of interaction type $r$. ${\mathcal{N}}_{i}^{r}$ is the neighbor set of node $v_{i}$ under interaction type $r$. $1/\surd \left| {{\mathcal{N}}_{i}^{r} } \right|\left| {{\mathcal{N}}_{j}^{r} } \right|$ and $1/\surd \left| {{\mathcal{N}}_{i}^{r} } \right|$ are normalization constants. $\sigma$ is a non-linear activation function like $ReLU$. The node features are initialized as one-hot vectors and input to the first layer, denoted as $h_{i}^{0} = x_{i}$. We stack two graph convolutional layers such that the final node embedding is computed as: $z_{i} = h_{i}^{K}$ with $K = 2$.

#### Subgraph perspective

Instead of taking the graph as a whole, we split the graph $G$ into two subgraphs, the binary association subgraph $\overline{G}$ (including the CC-graph, GG-graph, CP-graph, GP-graph) and multi-interaction subgraph $\tilde{G}$ (the CG-graph). We respectively use two 2-layer graph convolutional networks for learning node embedding in these two separate subgraphs.

In the binary association subgraph $\overline{G}$, chemical nodes only encode information from the neighbor nodes of chemicals and pathways, while gene nodes receive message from the neighbor nodes of genes and pathways. The hidden state $\overline{h}_{i}^{{\overline{k}}} \in {\mathbb{R}}^{{\overline{d}_{{\overline{k}}} }}$ of each hidden layer in the first 2-layer graph convolutional network is updated similarly as Eq. (1). The only difference is $r \in \overline{R}$. We assign the output node embedding as $\overline{z}_{i} = \overline{h}_{i}^{{\overline{K}}}$ with $\overline{K} = 2$. These embeddings are then transferred to the subgraph $\tilde{G}$ to initialize corresponding chemical and gene features, denoted as $\tilde{x}_{i} = \overline{z}_{i}$, where $v_{i} \in \{ V_{c} \cup V_{g} \}$.

As the observations in “Data observation” section suggest, we take account of extracting latent links to reconstruct the topological structures of nodes in the multi-interaction subgraph $\tilde{G}$. By searching over the entire graph $G$ with the substructure S-G-P, we screen out candidate latent links under each interaction type, denoted as $L_{r} = \left\{ {l_{i}^{r} } \right\}_{{i \in \left[ {N^{r} } \right]}}$, where $N^{r}$ is the number of candidate latent links under interaction type $r$. Let $\hat{N}_{i}^{r}$ denotes the number of substructures containing latent link $l_{i}^{r}$. A candidate latent link $l_{i}^{r}$ is decided to be the definite latent link if:

2 $$\hat{N}_{i}^{r} \ge \max \left( {2,\max \left( {\hat{N}_{0}^{r} ,\hat{N}_{1}^{r} , \ldots ,\hat{N}_{{N^{r} }}^{r} } \right) \times \lambda } \right),$$

where $\lambda$ is the threshold coefficient.

We use the confirmed latent links to update the topological properties of each node $v_{i}$. The set of new neighbors of node $v_{i}$ under interaction type $r$ can be denoted as ${\mathcal{L}}_{i}^{r}$. With taking account of the information propagated across latent edges, the hidden layer of the second 2-layer graph convolutional network is defined as follow:

3 $$\tilde{h}_{i}^{{\tilde{k} + 1}} = \sigma \left( {\mathop \sum \limits_{r} \left( {\mathop \sum \limits_{{j \in {\mathcal{N}}_{i}^{r} }} \frac{1}{{\sqrt {\left| {{\mathcal{N}}_{i}^{r} } \right|\left| {{\mathcal{N}}_{j}^{r} } \right|} }}\tilde{W}_{r}^{{\tilde{k}}} \tilde{h}_{j}^{{\tilde{k}}} + \mu^{r} \mathop \sum \limits_{{l \in {\mathcal{L}}_{i}^{r} }} \frac{1}{{\sqrt {\left| {{\mathcal{N}}_{i}^{r} } \right|\left| {{\mathcal{L}}_{l}^{r} } \right|} }}\tilde{W}_{r}^{{\tilde{k}}} \tilde{h}_{l}^{{\tilde{k}}} } \right)} \right),$$

where $\tilde{h}_{i}^{{\tilde{k}}} \in {\mathbb{R}}^{{\tilde{d}_{{\tilde{k}}} }}$ with $\tilde{d}_{{\tilde{k}}}$ denotes the dimensionality of the $\tilde{k}$-th hidden layer. $r \in \tilde{R}$ denotes one of the interaction types. Importantly note that $\mu^{r} \in \left[ {0,1} \right]$ is a trainable parameter, defined as latent rate, used to measure the contribution of latent links for interaction type $r$. The final node embedding is assigned as: $z_{i} = \tilde{h}_{i}^{{\tilde{K}}}$, where $\tilde{K} = 2$ and $v_{i} \in \{ V_{c} \cup V_{g} \}$.

### Tensor decomposition decoder

Given a chemical $v_{i}$ and a gene $v_{j}$, the decoder returns the probability ${\mathcal{P}}_{r}^{ij}$ of an edge $e_{ij} = \left( {v_{i} ,r,v_{j} } \right)$, which represents how likely chemical $v_{i}$ results in an interaction type $r$ of gene $v_{j}$. The decoder takes advantage of a tensor decomposition model, called DEDICOM [45], to formulate chemical-gene interactions, as shown in Fig. 7.

Based on the node embeddings $z_{i}$ and $z_{j}$ learned by the encoder, the decoder computes a score ${\mathcal{G}}\left( {z_{i} ,r,z_{j} } \right)$ for the edge $e_{ij}$, and then act a sigmoid function $\sigma$ on it as follow:

4 $${\mathcal{G}}\left( {z_{i} ,r,z_{j} } \right) = z_{i}^{T} {\mathcal{D}}_{r} {\mathcal{R}\mathcal{D}}_{r} z_{j} ,$$

5 $${\mathcal{P}}_{r}^{ij} = \sigma \left( {{\mathcal{G}}\left( {z_{i} ,r,z_{j} } \right)} \right),$$

where ${\mathcal{D}}_{r}$ is a local diagonal matrix giving weights to each dimension of the node embedding under interaction type $r$. ${\mathcal{R}}$ is a global parameter matrix associated with all interaction types, which enables the model to share information across different interaction types. Note that the matrix ${\mathcal{D}}_{r}$ and ${\mathcal{R}}$ are both trainable parameters of shape $d_{k} \times d_{k}$. These two matrices are initialized using the same method introduced in Glorot et al. [46].

### Model training

We perform negative sampling during the training procedure, which can reduce the training time greatly. We generate a negative sample $\left( {v_{i} ,r,v_{n} } \right)$ by replacing the node $v_{j}$ of the known edge $\left( {v_{i} ,r,v_{j} } \right)$ with node $v_{n}$, which is chosen randomly according to a sampling distribution in Mikolov et al. [47]. Specifically, the distribution probability of node $v_{n}$ is calculated based on its degree $d\left( {v_{n} } \right)$ as follow:

6 $$p\left( {v_{n} } \right) = \frac{{d\left( {v_{n} } \right)^{{{\raise0.7ex\hbox{$3$} \!\mathord{\left/ {\vphantom {3 4}}\right.\kern-\nulldelimiterspace} \!\lower0.7ex\hbox{$4$}}}} }}{{\mathop \sum \nolimits_{i = 0}^{{\mathcal{N}}} \left( {d\left( {v_{i} } \right)^{{{\raise0.7ex\hbox{$3$} \!\mathord{\left/ {\vphantom {3 4}}\right.\kern-\nulldelimiterspace} \!\lower0.7ex\hbox{$4$}}}} } \right)}},$$

Given a set of chemical-gene pairs and the labels, we encourage the model to enlarge the margin $m$ by minimizing the hinge loss function [48]:

7 $$\ell \left( {\Theta } \right) = \mathop \sum \limits_{{\left( {v_{i} ,r,v_{j} } \right) \in \tilde{R}}} \max \left( {0,{\mathcal{P}}_{r}^{in} - {\mathcal{P}}_{r}^{ij} + m} \right),$$

where ${\Theta }$ is a set of neural network parameters. ${\mathcal{P}}_{r}^{in}$ denotes the probability of the negative sample $\left( {v_{i} ,r,v_{n} } \right)$ associated with the known edge $\left( {v_{i} ,r,v_{j} } \right)$. With the hinge loss, any case where the difference is larger than the margin $m$ will not be penalty.

## Data Availability

The code files are available at: https://github.com/WebyGit/CGINet.

## Abbreviations

CGIs: Chemical-gene interactions
NLP: Natural language processing
DNN: Deep neural network
CNN: Convolutional neural network
RNN: Recurrent neural network
LSTM: Long short-term memory network
POS: Part of speech
KNN: K-nearest neighbor
SVM: Support vector machine
SMILES: Simplified molecular-input line-entry system
SPS: Structural property sequence
STITCH: Search Tool for InTeractions of Chemicals
CTD: Comparative Toxicogenomics Database
GCN: Graph convolutional network
AUROC: Area under the receiver-operating characteristic
AUPRC: Area under the precision-recall curve
AP@k: Average precision for the top-k identifications

## References

[1] Paul SM, Mytelka DS, Dunwiddie CT (2010). How to improve R&D productivity: the pharmaceutical industry's grand challenge. Nat Rev Drug Discov.

[2] Karimi M, Wu D, Wang Z (2019). DeepAffinity: interpretable deep learning of compound–protein affinity through unified recurrent and convolutional neural networks. Bioinformatics.

[3] Shi Y, Zhang X, Liao X (2013). Protein-chemical interaction prediction via kernelized sparse learning svm. Biocomputing.

[4] Li BQ, Niu B, Chen L (2013). Identifying chemicals with potential therapy of HIV based on protein-protein and protein-chemical interaction network. PLoS ONE.

[5] Chen L, Lu J, Huang T (2014). Finding candidate drugs for hepatitis C based on chemical-chemical and chemical-protein interactions. PLoS ONE.

[6] Lu J, Chen L, Yin J (2016). Identification of new candidate drugs for lung cancer using chemical–chemical interactions, chemical–protein interactions and a K-means clustering algorithm. J Biomol Struct Dyn.

[7] Cheng Z, Zhou S, Wang Y (2016). Effectively identifying compound-protein interactions by learning from positive and unlabeled examples. IEEE/ACM Trans Comput Biol Bioinform.

[8] Lung PY, He Z, Zhao T (2019). Extracting chemical–protein interactions from literature using sentence structure analysis and feature engineering. Database.

[9] Peng Y, Rios A, Kavuluru R (2018). Extracting chemical–protein relations with ensembles of SVM and deep learning models. Database.

[10] Sun C, Yang Z, Wang L (2020). Attention guided capsule networks for chemical-protein interaction extraction. J Biomed Inform.

[11] Lu H, Li L, He X (2019). Extracting chemical-protein interactions from biomedical literature via granular attention based recurrent neural networks. Comput Methods Programs Biomed.

[12] Corbett P, Boyle J (2018). Improving the learning of chemical-protein interactions from literature using transfer learning and specialized word embeddings. Database.

[13] Liu S, Shen F, Komandur Elayavilli R (2018). Extracting chemical–protein relations using attention-based neural networks. Database.

[14] Sun C, Yang Z, Su L (2020). Chemical-protein interaction extraction via Gaussian probability distribution and external biomedical knowledge. Bioinformatics (Oxford, England).

[15] Sun C, Yang Z, Luo L (2019). A deep learning approach with deep contextualized word representations for chemical–protein interaction extraction from biomedical literature. IEEE Access.

[16] Donald BR (2011). Algorithms in structural molecular biology.

[17] Morris GM, Huey R, Lindstrom W (2009). AutoDock4 and AutoDockTools4: automated docking with selective receptor flexibility. J Comput Chem.

[18] Tabei Y, Yamanishi Y (2013). Scalable prediction of compound-protein interactions using minwise hashing. BMC Syst Biol.

[19] Fang J, Li Y, Liu R (2015). Discovery of multitarget-directed ligands against Alzheimer’s disease through systematic prediction of chemical–protein interactions. J Chem Inf Model.

[20] Lee I, Keum J, Nam H (2019). DeepConv-DTI: prediction of drug-target interactions via deep learning with convolution on protein sequences. PLoS Comput Biol.

[21] Monteiro NRC, Ribeiro B, Arrais JP. Deep neural network architecture for drug-target interaction prediction. In: International conference on artificial neural networks. Springer, Cham (2019), p. 804–809

[22] Li S, Wan F, Shu H (2020). MONN: a multi-objective neural network for predicting compound-protein interactions and affinities. Cell Syst.

[23] Lee B, Zhang S, Poleksic A (2020). Heterogeneous multi-layered network model for omics data integration and analysis. Front Genet.

[24] Kuhn M, Szklarczyk D, Franceschini A (2012). STITCH 3: zooming in on protein–chemical interactions. Nucleic Acids Res.

[25] Davis AP, Grondin CJ, Johnson RJ (2019). The comparative toxicogenomics database: update 2019. Nucleic Acids Res.

[26] Luo Y, Zhao X, Zhou J (2017). A network integration approach for drug-target interaction prediction and computational drug repositioning from heterogeneous information. Nat Commun.

[27] Wu Z, Li W, Liu G (2018). Network-based methods for prediction of drug-target interactions. Front Pharmacol.

[28] Wan F, Hong L, Xiao A (2019). NeoDTI: neural integration of neighbor information from a heterogeneous network for discovering new drug–target interactions. Bioinformatics.

[29] Kingma DP, Ba J. Adam: a method for stochastic optimization; 2014. arXiv:1412.6980

[30] Abadi M, Barham P, Chen J et al. Tensorflow: a system for large-scale machine learning. In: 12th {USENIX} symposium on operating systems design and implementation ({OSDI} 16) (2016), p. 265–283

[31] Perozzi B, Al-Rfou R, Skiena S. Deepwalk: online learning of social representations. In: Proceedings of the 20th ACM SIGKDD international conference on knowledge discovery and data mining (2014), p. 701–710

[32] Grover A, Leskovec J. node2vec: scalable feature learning for networks. In: Proceedings of the 22nd ACM SIGKDD international conference on knowledge discovery and data mining (2016), p. 855–864 10.1145/2939672.2939754 PMC5108654 27853626

[33] Golub GH, Reinsch C. Singular value decomposition and least squares solutions. In: Linear algebra (Springer, Berlin 1971), p. 134–151

[34] Cai D, He X, Han J (2010). Graph regularized nonnegative matrix factorization for data representation. IEEE Trans Pattern Anal Mach Intell.

[35] Kipf TN, Welling M. Semi-supervised classification with graph convolutional networks (2016). arXiv:1609.02907

[36] Jiang K, Li K, Qin F (2011). Assessment of a novel β2-adrenoceptor agonist, trantinterol, for interference with human liver cytochrome P450 enzymes activities. Toxicol In Vitro.

[37] Slavov S, Stoyanova-Slavova I, Li S (2017). Why are most phospholipidosis inducers also hERG blockers?. Arch Toxicol.

[38] Abe H, Saito F, Tanaka T (2016). Developmental cuprizone exposure impairs oligodendrocyte lineages differentially in cortical and white matter tissues and suppresses glutamatergic neurogenesis signals and synaptic plasticity in the hippocampal dentate gyrus of rats. Toxicol Appl Pharmacol.

[39] Liang S, Liang S, Yin N (2019). Toxicogenomic analyses of the effects of BDE-47/209, TBBPA/S and TCBPA on early neural development with a human embryonic stem cell in vitro differentiation system. Toxicol Appl Pharmacol.

[40] Zitnik M, Agrawal M, Leskovec J (2018). Modeling polypharmacy side effects with graph convolutional networks. Bioinformatics.

[41] Parsons AB, Brost RL, Ding H (2004). Integration of chemical-genetic and genetic interaction data links bioactive compounds to cellular target pathways. Nat Biotechnol.

[42] Sun M, Zhao S, Gilvary C (2020). Graph convolutional networks for computational drug development and discovery. Brief Bioinform.

[43] Harada S, Akita H, Tsubaki M (2020). Dual graph convolutional neural network for predicting chemical networks. BMC Bioinform.

[44] Xu K, Li C, Tian Y, et al. Representation learning on graphs with jumping knowledge networks (2018). arXiv:1806.03536

[45] Papalexakis EE, Faloutsos C, Sidiropoulos ND (2016). Tensors for data mining and data fusion: models, applications, and scalable algorithms. ACM Trans Intell Syst Technol (TIST).

[46] Glorot X, Bengio Y. Understanding the difficulty of training deep feedforward neural networks. In Proceedings of the thirteenth international conference on artificial intelligence and statistics (2010), p. 249–256

[47] Mikolov T, Sutskever I, Chen K et al. Distributed representations of words and phrases and their compositionality. In: Advances in neural information processing systems (2013), p. 3111–3119

[48] Srebro N, Rennie J, Jaakkola TS. Maximum-margin matrix factorization. In: Advances in neural information processing systems (2005), p. 1329–1336

